# A Virtual Necropsy: Applications of 3D Scanning for Marine Mammal Pathology and Education

**DOI:** 10.3390/ani12040527

**Published:** 2022-02-21

**Authors:** Ellen M. Chenoweth, Josh Houston, Kathy Burek Huntington, Jan M. Straley

**Affiliations:** 1 College of Natural Sciences and Mathematics, University of Alaska Fairbanks, Fairbanks, AK 99775, USA; 2 Natural Sciences Department, School of Arts and Sciences, University of Alaska Southeast, Sitka Campus, Sitka, AK 99835, USA; jmstraley@alaska.edu; 3 Jfactory, 3D Division Sitka, Sitka, AK 99835, USA; josh@jfactory.xyz; 4 Alaska Veterinary Pathology Services, Eagle River, AK 99577, USA; avps.kbh@gmail.com

**Keywords:** outreach, 3D scanning, education, pathology, necropsy, anatomy, virtual learning, stranding

## Abstract

**Simple Summary:**

Most whale biologists spend their careers in boats, getting a glimpse at whales only when they come up to the surface to breathe or occasionally to feed. Being able to walk right up to a whale, and even look inside its body, offers scientists and stranding network volunteers a rare and meaningful opportunity to learn from whales at close range. On 14 March 2021, a female adult humpback whale was found dead on a beach near Sitka, Alaska. A team of volunteers performed a necropsy, meaning they dissected the whale to gather information about its cause of death and collected samples for further research (NOAA Fisheries permit 18786-01). Before, during and after the necropsy, the whale was three-dimensionally (3D) scanned using a drone and an iPad. These scans were annotated and arranged into a free publicallyavailable resource known as the 4D virtual necropsy (with time as the 4th dimension). After one month, we documented broad interest in this resource from researchers, educators, community members, and volunteers who respond to marine mammal strandings. We believe 3D scanning of future stranded animals will be useful for a wide range of applications.

**Abstract:**

Stranded large whales represent an opportunity to learn about the anatomy and health of these cryptic free-ranging animals. However, where time and access is frequently limited, law enforcement and management priorities often take precedence over research, outreach, and educational uses. On 14 March 2021, a dead female adult humpback whale was reported stranded on an uninhabited island 15 miles west of Sitka, Alaska. The whale was three-dimensionally scanned using light detection and ranging (LiDAR) and photogrammetry before, during, and at multiple time points after a necropsy, including full decomposition 17 days later (NOAA Fisheries permit 18786-01). These scans were organized and displayed on the site Sketchfab with annotations and made publically available as a “4D virtual necropsy” (the fourth dimension is time). After one month, our user survey indicated widespread interest in the platform by both the local community and worldwide by stranding professionals, researchers, and educators. We are unaware of another 3D scan involving a large whale with soft tissue for teaching, research, or public display, despite the ease of 3D scanning with current technologies and the wide-ranging applications.

## 1. Introduction

Necropsies of marine mammals are irreplaceable opportunities for informing marine mammal management, law enforcement, and research [1,2]. This is particularly true for free-ranging animals, including large cetaceans that are not studied in captivity. However, the quality of information obtained during a necropsy is restricted by how quickly the carcass is discovered, decomposition conditions, accessibility, the speed with which a team can be assembled, and the number and experience level of personnel able to assist and transport samples and data in good condition. The ability to gather a large experienced team often results in a direct trade-off with the speed of a response. Prioritizing research and management goals also limits the accessibility of the carcass for educational or community-engagement purposes, despite the impact of these experiences on participants, as evidenced by the large number of people who annually volunteer their time to participate in marine mammal stranding response [2,3,4].

Three-dimensional (3D) scanning is a process that collects data on the shape and appearance of an object or scene [5]. Depictions of this data allow for a more engaging, immersive and novel experience than photographs alone, as evidenced by their extensive use in the entertainment industry. Additionally, because they depict their subjects in a more realistic manner, they are thought to reduce cognitive load associated with the translation of two-dimensional (2D) depictions and to therefore facilitate learning [6,7]. Importantly for research applications, these data also retain the shape of the object including the absolute size, the relative position of features, and textures. Different methods of 3D capture, including light detection and ranging (LiDAR) and photogrammetry, have been developed and improved with different strengths and limitations. In addition, key advances in this technology continue to increase its accessibility. Currently, it is possible to capture 3D data using tablets and smartphones.

Recently, 3D technologies, which have been important in architecture, engineering, construction, and entertainment, have expanded into a wider range of applications with improvement in the ability of computers to process large datasets [8]. Artifacts, including whale fossils and skeletons of stranded whales [9], have been digitally scanned to increase access for education and research as well as document changes in condition [10]. The ability to three-dimensionally scan artifacts preserves information about them in their current condition and allows researchers and curators to track changes in their condition over time and increase their accessibility for research, education, and natural history. These goals are even more important in the case of a decomposing whale where changes in condition occur rapidly and accessibility is much more restricted than an artifact in a museum. The 3D modeling of small cetaceans has been used in education to demonstrate marine mammal anatomy and physiology is a simplified form [11].

Here, we present the first use of 3D scanning on the soft tissue of a large stranded cetacean to create a realistic virtual necropsy experience. We document the process of scanning a whale over a three-month period, including multiple scans during a necropsy. We then describe how additional materials have been added to create a user-friendly educational experience: the four-dimensional (4D) virtual necropsy (4D refers to the fact that sequential scans demonstrate change in the carcass over time). We present data to demonstrate the broad use of this currently unique resource and indicate a range of potential uses for this technology as applied marine mammals in the realms of education, research, diagnostic pathology, and management.

## 2. Materials and Methods

### 2.1. Field Necropsy

On 14 March 2021, a 13.4 m long female humpback whale was discovered by the US Coast Guard on Kruzof Island near the community of Sitka, Alaska. During the following days and three months, the whale was repeatedly three-dimensionally scanned using photogrammetry from a drone. The necropsy team was led by Dr. Lauren Wild, a research biologist and a professor of Fisheries Technology at the University of Alaska Southeast (UAS-Sitka), under the authorization of NOAA Fisheries.

Dr. Wild was assisted by nine experienced volunteers including a drone pilot/3D scanner. There was no veterinary pathologist on site. The whale was initially found in a supine position with its throat grooves inflated, indicating moderate decomposition. The whale was scanned using photogrammetry via drone. Four days later, the whale deflated and turned into a more prone position angled slighting onto its right side. The necropsy team was able to work on the whale from approximately 9 a.m. to 1 p.m. local time. The whale was scanned using photogrammetry and then measured. The upper and lower abdominal cavity, both shoulders, sections of the head, and thoracic cavity were exposed, including the removal of several ribs. Each of these sections of whale was photographed and scanned with LiDAR. The blubber was cut at approximately 0.5–1 m intervals along the length of the body to look for indications of trauma. Samples of blubber, baleen, stomach contents, one eyeball, and lung tissue were removed for further analysis. No broken bones were found, although the vertebrae and the majority of the skull were not exposed for examination. The samples and data were reviewed by Alaska Veterinary Pathology Services.

In addition, during a necropsy, the whale was also repeatedly scanned using LiDAR. Offsite, the photogrammetry data were processed into a textured mesh, measured and cropped in Blender™ Amsterdam, The Netherlands and uploaded to the website Sketchfab New York, NY, USA [12]. The 4D virtual Necropsy is hosted on a Google Sites™ Mountain View, CA USA page that is accessible through the University of Alaska website [13]. User information was collected through an anonymous survey.

### 2.2. 3D Scanning

**Figure 1:**
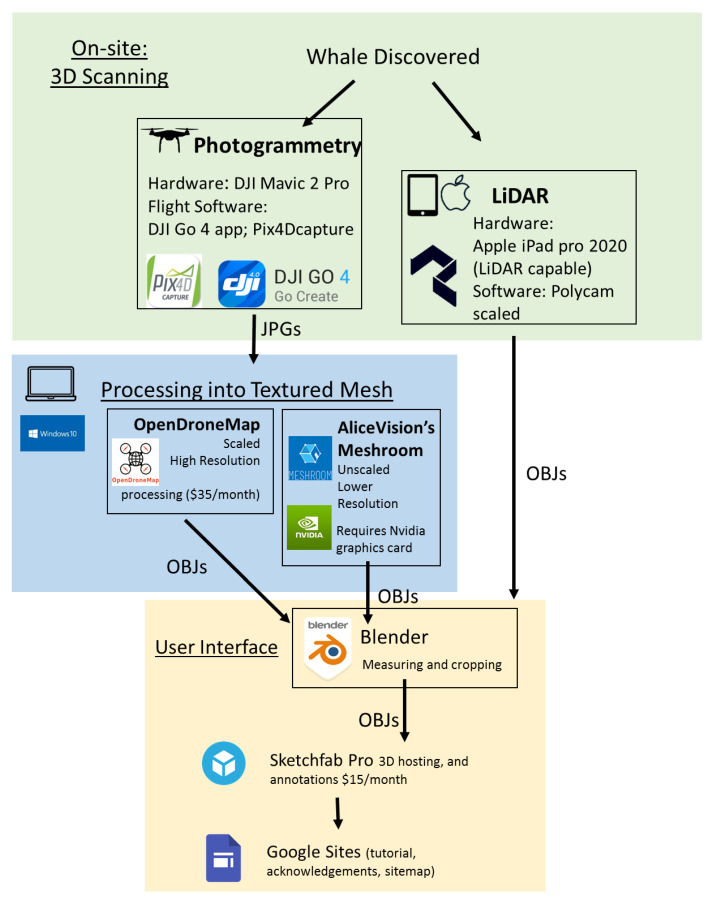
The data workflow that was used to generate a virtual necropsy experience from a stranded whale. After the whale was initially discovered, it was three-dimensionally scanned using both photogrammetry and light detection and ranging (LiDAR). While LiDAR software Polycam generated OBJ files in the field, photogrammetry produced ordered JPG files that were later assembled into a textured mesh using both OpenDroneMap and AliceVision^®^’s Meshroom. All OBJ files could then be further processed, edited and measured in Blender. Ultimately, these edited OBJ files were uploaded to the Sketchfab website. Within Sketchfab, annotations were added to provide logical navigation with the different scans and additional links to resources stored on Google Drive. Finally, a Google site was created to provide the context for the virtual necropsy experience including educational resources, a brief video user tutorial, and a site map with quick links to different aspects of the necropsy (main necropsy home: https://sites.google.com/alaska.edu/virtual-necropsy/home (last accessed on 15 February 2022); necropsy workshop home: https://sites.google.com/alaska.edu/virtual-necropsy-workshop/home) (last accessed on 15 February 2022). Here, “scaling” refers to scaling in absolute units. All digital reconstructions had the *x*, *y*, and *z* axes properly scaled relative to each other.

Two different 3D scanning techniques were used: photogrammetry and LiDAR, each with different strengths and limitations (Figure 1). Photogrammetry is done by collecting geo-referenced photographs, either simultaneously from different perspectives or in our case from a single platform (DJI Mavic 2 Pro drone using software DJI GO 4 app Nanshan, Shenzhen, China [14] and Pix4DCapture [15] Prilly, Switzerland), moving around an object. These photographs and their location data are then digitally processed into a textured mesh. This mesh consists of triangles outlining the surface of an object. The size, density and quantity of these triangles relate to the resolution of the shape surface render with higher densities of smaller triangles generating a more detailed and realistic shape, while lower densities create a more simplified boxier-looking shape. A texture, an image generated by the photographs, is then digitally applied to the mesh. The resolution of the texture is determined by the resolution of the photographs that generated it and whether those photographs depict the object from a variety of perspectives.

Photographs were processed both in AliceVision^®^’s Meshroom Los Angeles, CA, USA [16] and OpenDroneMap [17] (Figure 1). OpenDroneMap produces images at a higher resolution than Meshroom, but in many cases, the difference in the resolution was not meaningful for our purposes. The products of both OpenDroneMap and Meshroom are proportioned true to life (*x*, *y*, and *z* axes are in proportion to each other and the original object). OpenDroneMap additionally applies GIS coordinates to provide an absolute scale for the 3D mesh produced by the scan. In our case, we were able to use this OpenDroneMap data to apply scales to the Meshroom to produce reconstructions, except for in the case of a scan of a barnacle, where a scale of appropriate size was not available. The result of post-processing is OBJ files, an open geometry definition file format.

LiDAR allows for quicker scanning and field processing than photogrammetry. Similar to SONAR, which uses sound, LiDAR capable devices are able to emit light energy and receive reflected light. This generates a point cloud of colored pixels in 3D space that are programmatically interpolated in the field to generate a 3D mesh and texture. All the scans in this necropsy were gathered by one operator with a LiDAR capable Apple iPad Pro 2020 Cupertino CA, USA running the software Polycam San Jose CA, USA [18]. Recent advances in LiDAR processing with Polycam allow for realistic and uninterrupted scans of shiny objects, which was essential for this particular project due to the amount of water and wet surfaces scanned.

**Figure 2:**
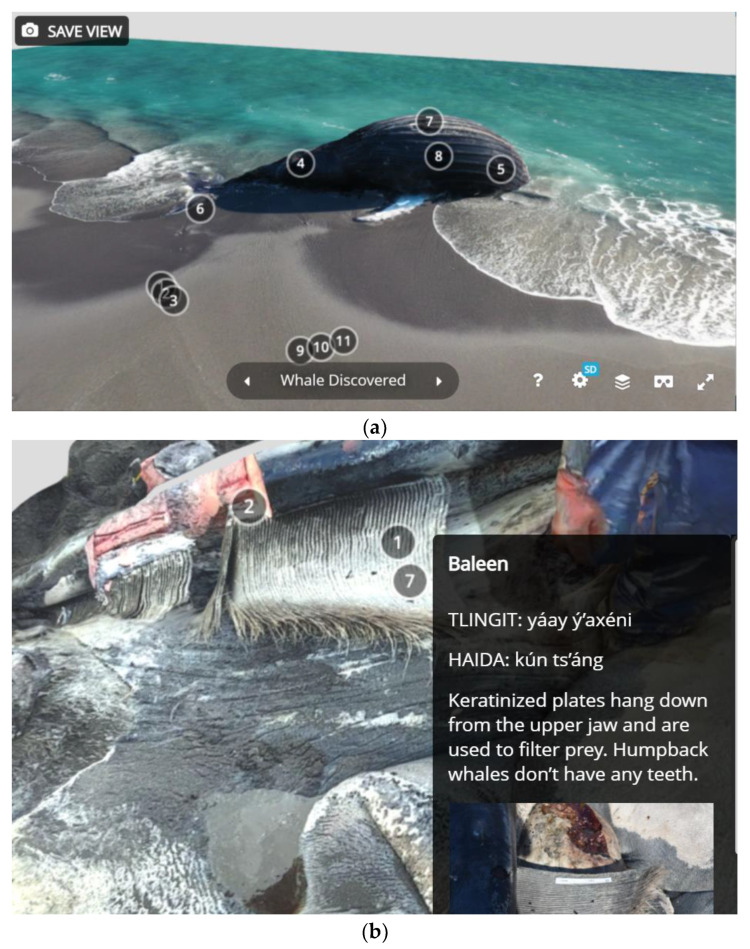

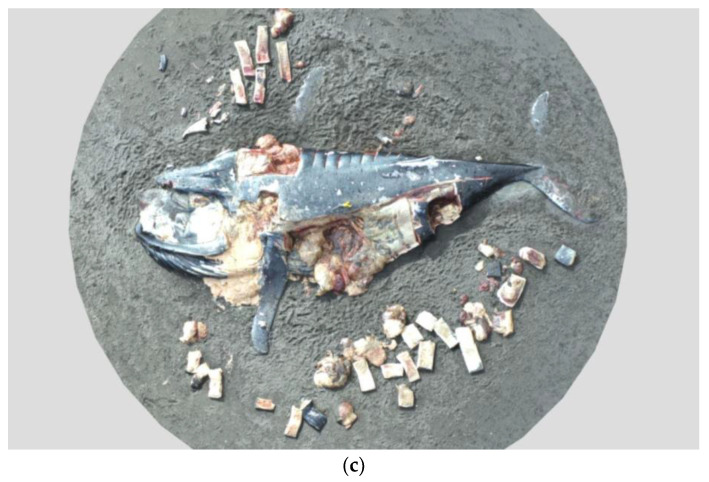
Images of the three-dimensional (3D) scans produced before (**a**), during (**b**) and after (**c**) the necropsy. These images show the scans with annotations, as they appeared in the four-dimensional (4D) virtual necropsy. Circled numbers indicate where additional annotations are available.

Whether OBJ files were generated with LiDAR or photogrammetry, they are editable in the program Blender [19] (Figure 1). Blender allows for cropping the mesh of the reconstructed object, managing its orientation and joining multiple scans. In one case, we scanned the sides of the whale at a high resolution with LiDAR but were not able to access the top of the whale with LiDAR. In Blender, we joined the texture from the photogrammetry scan to complete the reconstruction of the entire visible whale while preserving the higher-resolution texture on most of the reconstructed object. After post-processing, the edited OBJs were uploaded to the website Sketchfab, where users could view and interact with the edited and cropped digital reconstruction, changing their point of view and orientation and zooming in and out (Figure 2).

### 2.3. Assembling the Scans into the 4D Virtual Necropsy Experience

The scans were then ordered and linked via the annotation capability of the Sketchfab platform. A whale researcher who was present on site for the necropsy and a veterinary pathologist collaborated to interpret the information presented for both users without basic knowledge of whale biology as well as research professionals. In addition to providing interpretive information, these annotations also provided external links to resources such as photographs of the necropsy, research papers, and government websites. Annotation topics included practical information (e.g., what to do if you find a dead marine mammal), whale internal and external anatomy and physiology, marine adaptations, diagnostics (including signs of trauma, histology, sample collection, and parasitology), and cultural information specific to the Tlingit, Haida, and Tsimshian Indigenous peoples of Southeast Alaska [20,21,22]. In some cases, additional information from other necropsies was provided with permission, to demonstrate additional presentations of pathology or trauma not visible with this whale or in the scans obtained.

A workshop version of the necropsy was also created by modifying the main necropsy. This version was intended as an out-of-the box lesson for middle school, high school, or college students. Learning objectives include the following: (1) students will be able to identify threats to marine mammal health, including human threats; (2) students will be able to identify signs of normal healthy functioning and signs of pathology; and (3) students will practice data collection and use observations to develop scientific inference. Instructors are given a list of discussion questions to activate students’ relevant prior knowledge. Students are then told to imagine that they have arrived at the site of a dead whale and that they need to assess whether the whale is a good candidate for a necropsy. An initial assessment form is included that guides students through collecting and assessing relevant information. Then, students begin the necropsy and receive a similar form for assessing internal condition. Students are prompted to gather data in the form of photographs or screen shots to back up their assertions about the condition of the whale. Finally, instructors are provided with an outline of a post-necropsy debrief.

### 2.4. Disseminating the Necropsy and Assessing User Profiles

The virtual necropsy was made publicly available on 18 November 2021 via links on the University of Alaska Southeast’s website, although additions and edits will continue to be made. This release was coordinated with a press release. The necropsy link was also emailed to a list of potentially interested colleagues, including those present at the necropsy. We submitted the link to the MarMam listserve Victoria, B.C. Canada, a long-time resource for marine mammal researchers and managers that claims over 15,000 subscribers as of 2020 [23]. We submitted an invited article to the Alaska Marine Mammal Stranding Network Newsletter [24]. The virtual necropsy workshop was also incorporated into a dual-enrollment course at the University of Alaska Southeast (CRN: 77201: Current Topics in Marine Research) in the fall of 2021 with 45 enrolled students.

User information was gathered through a Google form on the main virtual necropsy Google site. Users who clicked to enter the necropsy were redirected to a short survey (see Supplementary Information). The full survey is included in Supplementary Materials. It is possible to circumvent the survey through links in the sitemap or by logging on with another user, for example when users accessed the necropsy in a group. Another Google form was included as an optional feedback form at multiple times during the necropsy. However, this form was not widely utilized (fewer than 20 responses), and the results are therefore not included here. A form is not included in the workshop version of the necropsy.

## 3. Results

The virtual necropsy consists of a total of 24 unique original scans offered in two different formats: the open-ended full necropsy format and the more structured workshop format, which takes approximately 90 minutes to complete. Users can click through annotations in order for a guided experience from discovering and whale through the necropsy and decomposition. Alternatively, users can click areas of interest and explore on their own. The virtual necropsy is publically available and free. The individual scans are downloadable as well (Table 1). While the cause of death was not conclusive, the presence of bloody fluid upon cutting through the blubber and muscle and condition of the muscle on the right dorsal side of the whale could be consistent with hemorrhage resulting from trauma, perhaps from a ship strike. However, this was not conclusive due to the state of decomposition. The whale otherwise appeared to be in good condition with a healthy-looking blubber layer and muscle, low parasite load, and partially digested fish found in the digestive tract.

**Figure 3:**
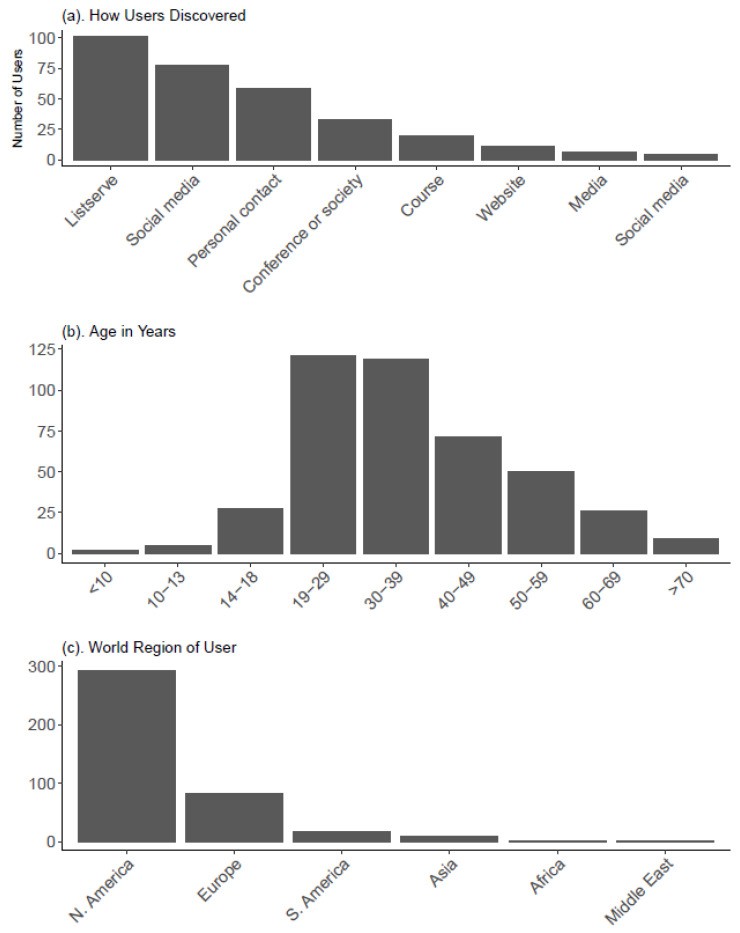

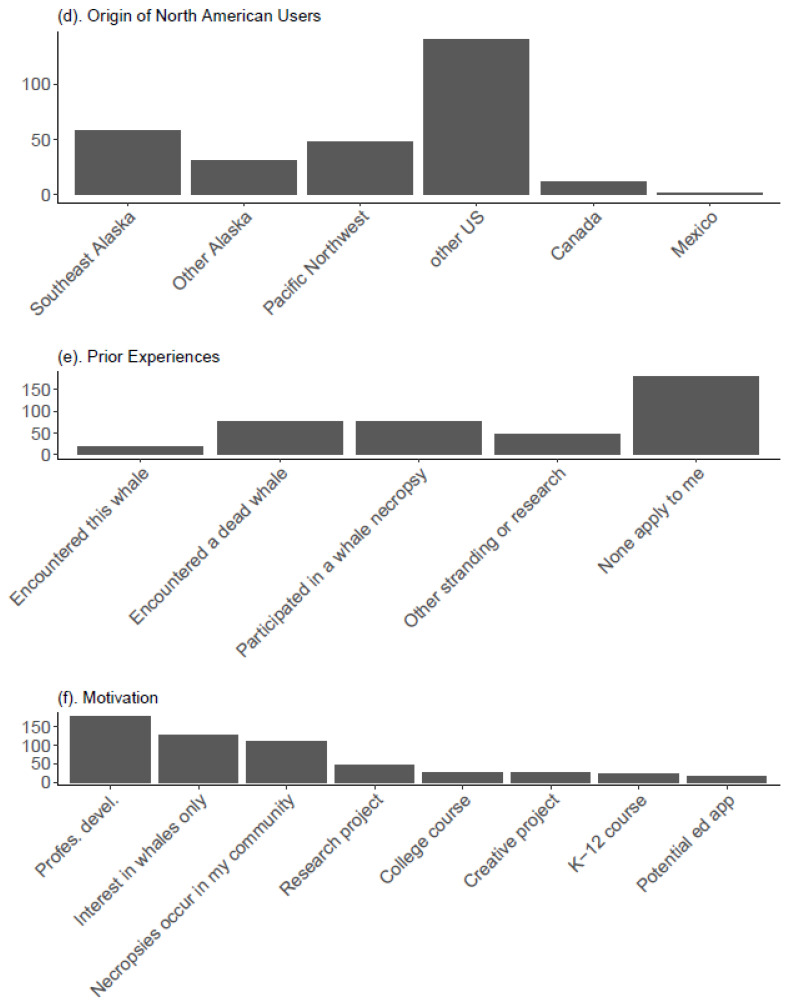
Virtual necropsy user pre-survey results. Users accessing the necropsy through the main Google site were redirected to an anonymous Google survey prior to accessing the first 3D scan. Users were free to skip any questions. The results shown here summarize survey questions and results. The full questions with answer options are provided in Supplementary Materials.

As of 18 December 2021, the virtual necropsy has been publicly available for one month. There were 485 user responses received, with 90.7% responding that they were first-time users. In the first month of availability, our 4D virtual necropsy was accessed over 400 times from 37 countries or territories (Figure 3). The users most commonly cited a listserve in the way they learned about the virtual necropsy. The virtual necropsy was submitted to the popular Marmam listserve [23]. The users were most commonly between 18 and 40 years old that tended to coincide with higher education levels and users earlier in their careers. Accordingly, professional development was the most common motivation for accessing the necropsy. However, the second most common motivation was just general interest. A general audience was also reflected in a large number of users from Southeast Alaska or who noted that whale necropsies occur in their communities. Other common motivations included taking a course, research applications, other creative projects, or an interest in potentially using the necropsy as an educational resource in the future. This group included K-12 educators, outreach specialists, university professors, and stranding coordinators.

## 4. Discussion

We have demonstrated that a dead whale can be scanned in the field with minimal dedication of human resources and time. The result was a virtual experience that preserved some information from this stranded animal and increased the accessibility of that information to scientists, managers, and citizens to information about this animal and the necropsy experience more generally. The swift dissemination of this resource to local, national, and international users reinforces the diverse applications of this technology.

Scanning caused minimal delays in the overall progress of the necropsy, similar to standard photography. The drone scans of the entire whale were completed in less than 15 min, while other necropsy volunteers were donning gear, unpacking equipment, and supplies, organizing the site or still arriving on the scene. The use of one operator was important, since the site was accessed by helicopter, limiting the number of personnel that could participate in the necropsy. In other situations, an additional observer would be useful for drone work to monitor the scene while the pilot is flying. A LiDAR scan of all but the very top of the whale was accomplished in less than 10 min.

3D scanning technology is becoming increasingly accessible to the non-expert user. There are now iPhones (12) that are LiDAR-enabled. In Alaska, dead stranded animals are often first reported by fisherman, beachcombers, or other community members. With only a cell phone, these individuals could be directed to three-dimensionally scan it and send the information to a pathologist or stranding coordinator to conduct an initial assessment. This scan would provide the context, scale, and imagery necessary for a thorough initial assessment and to plan a necropsy response. The community member would not need to touch the specimen or interact with it any more closely than to photograph it. LiDAR scans do not require post-processing and can be assessed in the field for general completeness and adequacy. Compared to LiDAR, we found that photogrammetry is well suited for smaller objects. For example, it was used to create a 3D model of a whale barnacle. However, it was also used to generate full whale scans due to the ability to scan with photogrammetry via drone without going ashore or to generate data in areas of the whale that were not accessible from a hand-held iPad (i.e., the top of the whale).

We have already demonstrated how the necropsy can be used more generally for educational applications. While simplified models are helpful for teaching concepts [3], realistic depictions can be used to teach more general understanding of uncertainty and ambiguity inherent in field science. We anticipate this resource will be used to prepare volunteers for assisting more capably with a live necropsy.

3D scanning is also useful to allow access for specialists who cannot access a carcass or cannot access it immediately. While many necropsies are attended by veterinarians and/or veterinary pathologists, this is not always possible, including the case of this necropsy. The inclusion of 3D imagery in addition to still photographs and tissues samples from the site allows a more accurate assessment of the animal by specialists after the fact. The added context and scale of a 3D scan could be decisive in determining the cause of death, maximizing the information from a remote specimen, capturing evidence when the whale is at its most fresh or more thoroughly documenting the scene for use in law enforcement.

3D scanning of dead marine mammals could provide important data for future biological research. Previous work with 3D modeling of whales from stranding measurements has shown how more detailed reconstructions can produce better results for surface area-to-volume ratios, which are important in thermoregulation modeling [25]. Using 3D scans of fresh stranded animals could improve these models further with implications for hydrodynamics and energetics modeling. Although this particular whale was misshapen by decomposition, a fresher whale might provide the opportunity to also scan internal organs and structures where accurate surface area and volume measurements have important implications for internal physiological processes. Due to their superlative size, allometric scaling of whales is a topic of interest for evolutionary biology [26,27]. We also demonstrated how 3D modeling can be useful in documenting the decay of a carcass. Combined with more specimens and information about local environmental conditions, 3D scanning could be useful for the study of taphonomy to infer the environmental conditions present during the deposition of fossilized remains [28,29].

## 5. Conclusions

The 4D Virtual Necropsy remains an open-access resource that increases access to the rare experience of a large whale necropsy for a variety of applications. Public access to these materials helps generate interest in marine mammal welfare and demystify these protected species that few people are able to experience first-hand. More generally, the 3D scanning of marine animals by citizen scientists could preserve information about animals as soon as possible during the decomposition process. 3D scanning during a necropsy can provide information to experts that are not on site to address a wide range of research questions.

## Figures and Tables

**Table 1:** Each of the main scans that served as a navigational hub for other sub-scans is listed here in a larger font (“Discovering a dead whale”, “Basic external anatomy”, “Whale necropsy”, “Post-necropsy”, “Decomposition 1”, “Decomposition 2”, and “Decomposition 3”). The black smaller font was used to represent sub-scans that are linked to and link back to the main scans while the small blue font was used to indicate external links to other resources.

Part 1: Site Assessment	Part 2: Necropsy (Looking Inside)	Part 3: Decomposition
Discovering a dead whale (14 March 2021) ▪ Level A assessment ▪ Whale location ▪ Species identification guide ▪ Taking measurements ▪ Photographic identification ▪ Reporting Basic external anatomy ▪ Disentanglement training ▪ Head and mouth ▪ Blowhole ▪ Eyeball ▪ Barnacles Site assessment photo album	Whale necropsy ▪ Getting started: dorsal side ▪ Deep Into A cut measure blubber thickness ▪ Right side ▪ Right side flayed open ▪ Lower abdomen 1 ▪ Lower abdomen 2 ▪ Upper abdomen ▪ Left side flayed open ▪ Thoracic cavity 1 ▪ Thoracic cavity 2 ▪ Behind the left shoulder ▪ Right shoulder Necropsy photo album	▪ Post-necropsy (18 March 18 2021) ▪ Decomposition 1 (3 April 2021) ▪ Decomposition 2 (18 April 2021) ▪ Decomposition 3 (13 June 2021) Whale skeleton (different whales)

## Data Availability

Not applicable.

## Supplementary Materials

The following supporting information can be downloaded at: https://www.mdpi.com/article/10.3390/ani12040527/s1. Virtual Necropsy User Information Survey.
