# Supplementary material for: A Virtual Necropsy: Applications of 3D Scanning for Marine Mammal Pathology and Education
Source: Animals (Basel). 2022 Feb 21;12(4):527. doi: 10.3390/ani12040527 (PMC8868430; doi:10.3390/ani12040527)
Supplement: Supplementary file 1 [file animals-12-00527-s001.zip › animals-1561797-supplementary.pdf]

Virtual Necropsy User Information Survey:

User instructions: Before you access the virtual necropsy, let us know a bit about you. Your answers may be used in a summary of who uses the whale necropsy and how they use it. We will do so without using any identifiable information.

Question 1: Is this your first time accessing the virtual necropsy?

- Yes
- No

If user responds "No", they are redirected to the completed survey page with a link to the necropsy, if they answer "Yes" they continue on to the follow questions.

Question 2 (short answer): How did you hear about the virtual whale necropsy?

Question 3 (multiple choice): How old are you?

- under 10 years old
- 10-13 years old
- 14-18 years old
- 19-29 years old
- 30-39 years old
- 40-49 years old
- 50-59 years old
- 60-69 years old
- over 70 years old

Question 4 (check boxes) Why are you accessing the virtual necropsy, check all that apply

- This is part of a middle school course
- This is part of a high school course
- This is part of a college course
- I am curious about this because whale necropsies sometimes occur in my community
- I am interested in whales
- I believe this will help me in my career or professional development
- I believe this may be useful for a research project
- I believe this may be useful for a creative or artistic project
- Other…

Question 5: Where do you live? Mark all that apply

- I currently or at one time lived in Southeast Alaska

- I currently or at one time lived in Alaska
- I live in the Pacific Northwest
- I live in the United States
- Other…

Question 6: Which of these experiences apply to you?

- I have worked or volunteered with the NOAA stranding network on a dead whale
- I have worked or volunteered with the NOAA stranding network on other marine mammals
- I have encountered a dead whale
- I encountered or worked on this dead whale
- None apply to me
- Other…
